# Phylogeny-wide analysis of G-protein coupled receptors in social amoebas and implications for the evolution of multicellularity

**DOI:** 10.12688/openreseurope.15250.1

**Published:** 2022-12-06

**Authors:** Grant Hall, Sarah Kelly, Pauline Schaap, Christina Schilde

**Affiliations:** 1 School of Life Sciences, University of Dundee, Dundee, DD1 5EH, UK; 2 Centre for Inflammation Research, University of Edinburgh, Edinburgh, EH16 4TJ, UK

**Keywords:** G-protein coupled receptor, dictyostelids, social amoebas, multicellularity, signalling, Amoebozoa

## Abstract

G-protein coupled receptors (GPCRs) are seven-transmembrane proteins and constitute the largest group of receptors within eukaryotes. The presence of a large set of GPRCs in the unicellular Amoebozoa was surprising and is indicative of the largely undiscovered environmental sensing capabilities in this group. Evolutionary transitions from unicellular to multicellular lifestyles, like we see in social amoebas, have occurred several times independently in the Amoebozoa, and GPCRs may have been co-opted for new functions in cell-cell communication.

Methods

We have analysed a set of GPCRs from fully sequenced Amoebozoan genomes by Bayesian inference, compared their phylogenetic distribution and domain composition, and analysed their temporal and spatial expression patterns in five species of dictyostelids.

Results

We found evidence that most GPCRs are conserved deeply in the Amoebozoa and are probably performing roles in general cell functions and complex environmental sensing. All families of GPCRs (apart from the family 4 fungal pheromone receptors) are present in dictyostelids with family 5 being the largest and family 2 the one with the fewest members. For the first time, we identify the presence of family 1 rhodopsin-like GPCRs in dictyostelids. Some GPCRs have been amplified in the dictyostelids and in specific lineages thereof and through changes in expression patterns may have been repurposed for signalling in multicellular development.

Discussion

Our phylogenetic analysis suggests that GPCR families 1, 2 and 6 already diverged early in the Amoebozoa, whereas families 3 and 5 expanded later within the dictyostelids. The family 6 cAMP receptors that have experimentally supported roles in multicellular development in dictyostelids ( *carA-carD*; *tasA/B*) originated at the root of all dictyostelids and only have weakly associated homologs in *Physarum polycephalum.* Our analysis identified candidate GPCRs which have evolved in the dictyostelids and could have been co-opted for multicellular development.

## Introduction

G-protein coupled receptors also known as seven-transmembrane receptors (7TMR) or serpentine receptors, comprise the largest class of conserved eukaryotic transmembrane proteins and are involved in sensing extracellular signals, such as odorants and signals used for cell-cell communication ( Bockaert & Pin, 1999). They perform various environmental sensing and signalling functions using heterotrimeric G-proteins as mediators. GPRCs of vertebrates are important regulators during development and tissue homeostasis and the over 800 human members are the most researched drug targets ( Insel *et al.*, 2019; Yang *et al.*, 2021). The seven transmembrane helices are the core unit of all GPCRs and they can have additional extracellular *N*-terminal sensing and *C*-terminal intracellular domains. Some GPCRs act as homo- or heterodimers and also associate with a variety of other membrane proteins ( Bockaert & Pin, 1999). Ligand binding to the extracellular domain, or in case of the rhodopsin-like family within the transmembrane helices, results in intramolecular changes in the intracellular loops 2 and 3 which activates the GPCR’s ability to exchange GDP for GTP and thereby activate the associated G proteins. The G-protein complex then dissociates into a G-alpha unit and a G-beta/gamma dimer, which can each activate downstream proteins, such as adenylyl cyclases, phospholipase C, ion channels, kinases, Rho-family guanine nucleotide exchange factors and other small G-proteins ( McCudden *et al.*, 2005). Because the effects of many hormones, neurotransmitters and neuropeptides are mediated by GPCRs, they are important targets for drug development.

Based on sequence similarity, GPCRs have been subdivided into six families ( Alexander *et al.*, 2019; Bockaert & Pin, 1999; Foord *et al.*, 2005; Hu *et al.*, 2017). A more recent phylogenetic study also recognises more atypical GPCRs like GPR108 and the intimal thickness-related receptor (ITR) as universal eukaryote GPCRs based on their HMM (hidden Markov model) predictions ( Nordstrom *et al.*, 2011). The Rhodopsin-like family 1 GPCRs is the largest group comprising several subfamilies of hormone, neuropeptide, neurotransmitter, nucleotide and light receptors. The family 2 secretin-like receptors are activated by glucagon-/secretin-like hormones and are related to the adhesion family GPCRs, which differ by containing a large cleavable *N*-terminus ( Alexander *et al.*, 2019). Family 3 comprises the metabotropic glutamate receptors. GPCRs of family 4 are pheromone mating factor receptors, exclusively found in fungi. The family 5 frizzled/smoothened GPCRs have widespread signalling roles in animal development and adult tissue homeostasis. The dictyostelid cAMP receptors comprise family 6 and have no orthologues outside this group.

Dictyostelid social amoebas are multicellular members of the otherwise mostly unicellular Amoebozoa, a sister group to the Opisthokonta, which comprise fungi and and animals. Dictyostelia are phylogenetically divided into two main branches each consisting of two major groups and some minor group intermediates ( Sheikh *et al.*, 2015). Branch I consists of group 1 (Cavenderiaceae) and group 2 (Acytosteliaceae) whereas branch II comprises groups 3 (Raperosteliaceae) and 4 (Dictyosteliaceae) ( Sheikh *et al.*, 2018). Dictyostelids display a different type of multicellularity than animals, fungi and plants becoming multicellular by aggregation of individual cells. Dictyostelid amoebas feed on bacteria as single cells but survive starvation by aggregating and forming asexual multicellular fruiting bodies (sorocarps) that can consist of up to five different cell types. The two main cell types, spore and stalk cells, are present in all dictyostelid families except the Acytosteliaceae. Whereas the unicellular spores can survive harsh environmental conditions for a long time and are the unit of dispersal for dictyostelids, stalk cells undergo altruistic cell death in the process of fruiting body formation. Differentiation into prespore and prestalk cells starts shortly after aggregation, but terminal encapsulation of spore and stalk cells occurs only during fruiting body formation. Like most Amoebozoa, dictyostelid amoebas live as unicellular bacteriophagic amoebas in soil. This requires a certain repertoire of environmental sensing receptors for finding food bacteria, avoiding predation and other stresses, and to find mating partners. Having inherited a repertoire of GPCRs from their last unicellular ancestor, it is conceivable that this was adapted and expanded during dictyostelid evolution to enable communication between cells during the multicellular phase. When Dictyostelid amoebas starve or are stressed, some species will encyst individually just like many other free-living amoebas. More commonly, deprivation of food will trigger aggregation and multicellular development, which all known dictyostelid species are capable of. The chemoattractant during aggregation is cAMP for most species in group 4 and glorin for some in groups 1 and 2 ( Romeralo *et al.*, 2013), while some group 3 species use folate or pterin. cAMP is involved in morphogenesis of all dictyostelids and the cAMP receptors represent GPCR family 6. Genomes are available for *Dictyostelium* species that represent the four major groups and for several related solitary Amoebozoa. For some Dictyostelia stage- and cell-type specific transcriptomes are also available. In this study we have used these data to assess conservation and change in the complete repertoire of GPCR genes across Dictyostelia and to investigate changes in developmental expression and functional domain architecture in the genes in the course of dictyostelid evolution.

## Methods

### Data retrieval

An InterProScan (RRID:SCR_005829) ( Jones *et al.*, 2014) was carried out of all transmembrane proteins from the published proteomes of *Dictyostelium discoideum* (DDB) ( Eichinger *et al.*, 2005), *Dictyostelium purpureum* (DPU) ( Sucgang *et al.*, 2011), ( Glöckner *et al.*, 2016), *Polysphondylium pallidum* (PPL), *Dictyostelium fasciculatum* (DFA) ( Heidel *et al.*, 2011), *Acanthamoeba castellanii* (ACA) ( Clarke *et al.*, 2013), *Physarum polycephalum* (Phypo) ( Schaap *et al.*, 2015) and *Protostelium aurantium var.fungivorum* (PROFUN) ( Hillmann *et al.*, 2018). G-protein coupled receptors were identified by their signature InterProScan GPCR domains. Comparison with earlier studies of GPCRs in some of the species revealed that they had not captured all family members. Representative members of each family were also used as bait in BLASTp (RRID:SCR_001010) searches of the eight Amoebozoan genomes to complete the dataset.

All GPCR sequences were aligned with Clustal Omega (RRID:SCR_001591) ( Sievers *et al.*, 2011) and a pilot tree was inferred by RAxML (RRID:SCR_006086) ( Stamatakis, 2014). This tree subdivided the full set into five major clades representing each GPCR family. New alignments and preliminary phylogenetic trees were generated for each clade with MrBayes 3.2.7 (RRID:SCR_012067) ( Ronquist *et al.*, 2012). These trees were scrutinised for any missing members of otherwise orthologous sets, and further BLASTp or tBLASTn (RRID:SCR_011822) searches were performed with a member of the set as bait. This yielded in some cases incorrectly predicted gene models, which were repaired guided by orthologous sequences, before final phylogenetic trees were inferred. 

### Phylogenetic analysis and tree annotation

The sequences of the 7TM domains plus 20 amino acids of flanking sequence were aligned with Clustal Omega ( Sievers *et al.*, 2011), large gaps and regions of poorly aligned sequence were deleted with Jalview 2.11.1.3 (RRID:SCR_006459) ( Waterhouse *et al.*, 2009) or BioEdit 7.0.5.3 (RRID:SCR_007361) ( Hall, 1999) and phylogenies were inferred using RAxML with 100 bootstrap replicates or using MrBayes with a mixed amino acid model and gamma distribution for rate variation between sites. Analyses were continued until the SD of split frequencies was <0.01 or up to 3,000,000 generations. The trees were mid-point rooted in FigTree v1.4.4 (RRID:SCR_008515) (A.Rambaut, University of Edinburgh, UK) and exported as .pdf files to Adobe Illustrator (RRID:SCR_010279) for further annotation.

Domain signatures were obtained using SMART (RRID:SCR_005026) ( Schultz *et al.*, 1998). To avoid automatic retrieval of old models, we replaced the start methionine by another amino acid. In case of overlapping domains, the domains with the lowest e-value or largest coverage were selected. The domain architectures were saved as a .svg files and placed next to their corresponding locus tags onto the phylogenetic trees.

Developmental and cell-type specific expression data were retrieved from published RNAseq experiments of the five Dictyostelid species ( Glöckner *et al.*, 2016; Kin *et al.*, 2018; Parikh *et al.*, 2010). Data were normalised as fraction of the maximum transcript read count for the developmental profiles (as in ( Forbes *et al.*, 2019)) and as fraction of the summed read counts for the purified cell types. Expression value sums of 10 or less per experiment were highlighted by 30% opacity. Expression data was combined for each orthologous clade and shown as a heat map. Where no expression data could be found the row was left blank. This happened frequently when gene models had changed between different expression experiments. Where expression data was available, but the new gene model indicated the presence of an earlier gene fusion, the data was omitted because expression reads were not re-mapped and could not be attributed unequivocally to the either of the fused genes.

## Results and discussion

**Figure 1.**
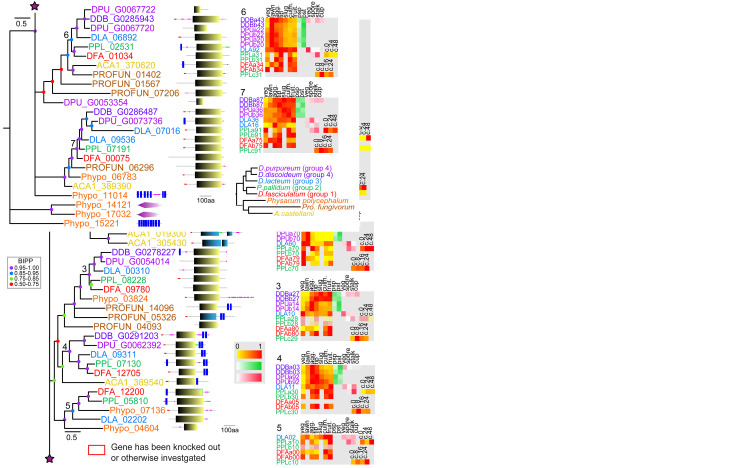
The family 1 rhodopsin-like GPCRs. Family 1 GPCRs were identified from InterproScan of the 8 Amoebozoan proteomes by the presence of InterproIDs IPR019336 or IPR009637 and further BLAST searches with rhodopsin-like receptors. Sequences were aligned and the phylogeny was inferred by Bayesian analysis. The scale bar indicates number of changes per site. Large trees have been split up and should be joined at the star signs. Locus tags are colour coded according to the species phylogenetic position (panel bottom right) and posterior probabilities of tree nodes (BIPP) are indicated by coloured dots. The locus tags are annotated with the SMART domain architecture of the protein shown next to it. Relative developmental stage and cell-type specific expression data for orthologous dictyostelid genes were presented as set of heatmaps for each clade, with transcriptional profiles for each gene identified by the first three and last two digits of the locus tag and ‘a’ and ‘b’ signifying replicate experiments. Abbreviations of developmental stages: veg.: vegetative; lawn: starving cells, agg.: aggregation; tip: tipped mounds; slug: migrating slugs; culm.: early to mid-fruiting bodies; fruit.: complete fruiting bodies; c.0 – c.48: hours into encystation, psp: prespore and pst: prestalk cells, vegetative (veg), spore, stalk and cup cells. The colour legend beside the heat maps indicates the level of expression (yellow-red: (developmental expression): fraction of maximum value, white-green (pre-cell type expression) and white-red (cell-type specific expression): fraction of summed reads). For genes with maximal read counts <10, the heat map is shown at 30% opacity. The normalised transcript counts were obtained from published data ( Glöckner *et al.*, 2016; Kin *et al.*, 2018; Parikh *et al.*, 2010). Note that not all stage and cell-type data is available for all species. Genes with known functions are boxed red and assigned names for *D.dis* genes are shown in purple italic font. *D.dis* locus tags for genes that result from the strain AX4 specific chromosome 2 duplication are indicated by 50% opacity.

The proteomes of the dictyostelids *Dictyostelium discoideum* (DDB) ( Eichinger *et al.*, 2005), *Dictyostelium purpureum* (DPU) ( Sucgang *et al.*, 2011), *Dictyostelium lacteum* (DLA) ( Glöckner *et al.*, 2016), *Polysphondylium pallidum* (PPL) and *Dictyostelium fasciculatum* (DFA) ( Heidel *et al.*, 2011) and the solitary amoebozoans *Acanthamoeba castellanii* (ACA) ( Clarke *et al.*, 2013), *Protostelium fungivorum* (PROFUN) ( Hillmann *et al.*, 2018) and the related myxogastrid *Physarum polycephalum* (PHYPO) ( Schaap *et al.*, 2015) were subjected to Interproscan ( Jones *et al.*, 2014) to identify their repertoires of protein functional domains. Proteins in the six classes of GPCRs were first identified by the Interpro identifiers of each class. Comparison with earlier studies of GPCRs in some of the species ( Eichinger *et al.*, 2005) ( Heidel *et al.*, 2011) revealed that these data sets were incomplete since the earlier studies had not captured all family members. Further BLASTp searches with the most diverged members of each class were performed to identify any missing proteins. A total of 414 non-redundant GPCRs were identified ( Table 1), which were assigned to the GPCR class for which their Interpro domains showed the lowest E-value and for cases where this was equivocal according to their phylogenetic affinity. No family 4 fungal pheromone GPCRs were found. Separate phylogenetic trees were prepared for each family, which were annotated with protein functional domains, and, for the dictyostelids, with heatmaps of developmental- and cell type- specific gene expression ( Glöckner *et al.*, 2016; Kin *et al.*, 2018; Parikh *et al.*, 2010) ( Figure 1– Figure 6).

**Table 1.** List of the number of proteins found per family and per species. Note that family 4 contains only fungal pheromone receptors and is not present in Amoebozoa. Outliers in numbers compared to the whole set were identified and are indicated in bold italic numbers. * Clade numbers only refer to clades with Dictyostelid members. In brackets are previously reported gene numbers from *D.dis* ( Prabhu & Eichinger, 2006)/( Nordstrom *et al.*, 2011) and *A.cas* ( Clarke *et al.*, 2013).

species	Family 1	Family 2	Family 3	Family 4	Family 5	Family 6
*A.cas*	5 (8)	4 (5)	***0*** (0)	-	16 (17)	7 (6)
*P.fun*	8	1	***0***	-	0	5
*Phy.pol*	9	4	***37***	-	***35***	***39***
*D.dis*	10 (0/[3])	3 (2/1)	17 (17/15)	-	27 (27/25)	12 (13/12)
*D.pur*	***13***	3	11	-	15	9
*D.lac*	8	2	10	-	8	8
*P.pal*	9	3	15	-	8	12
*D.fas*	8	2	7	-	14	11
						
clades *	7	2	11	-	8	10

**Figure 2.**
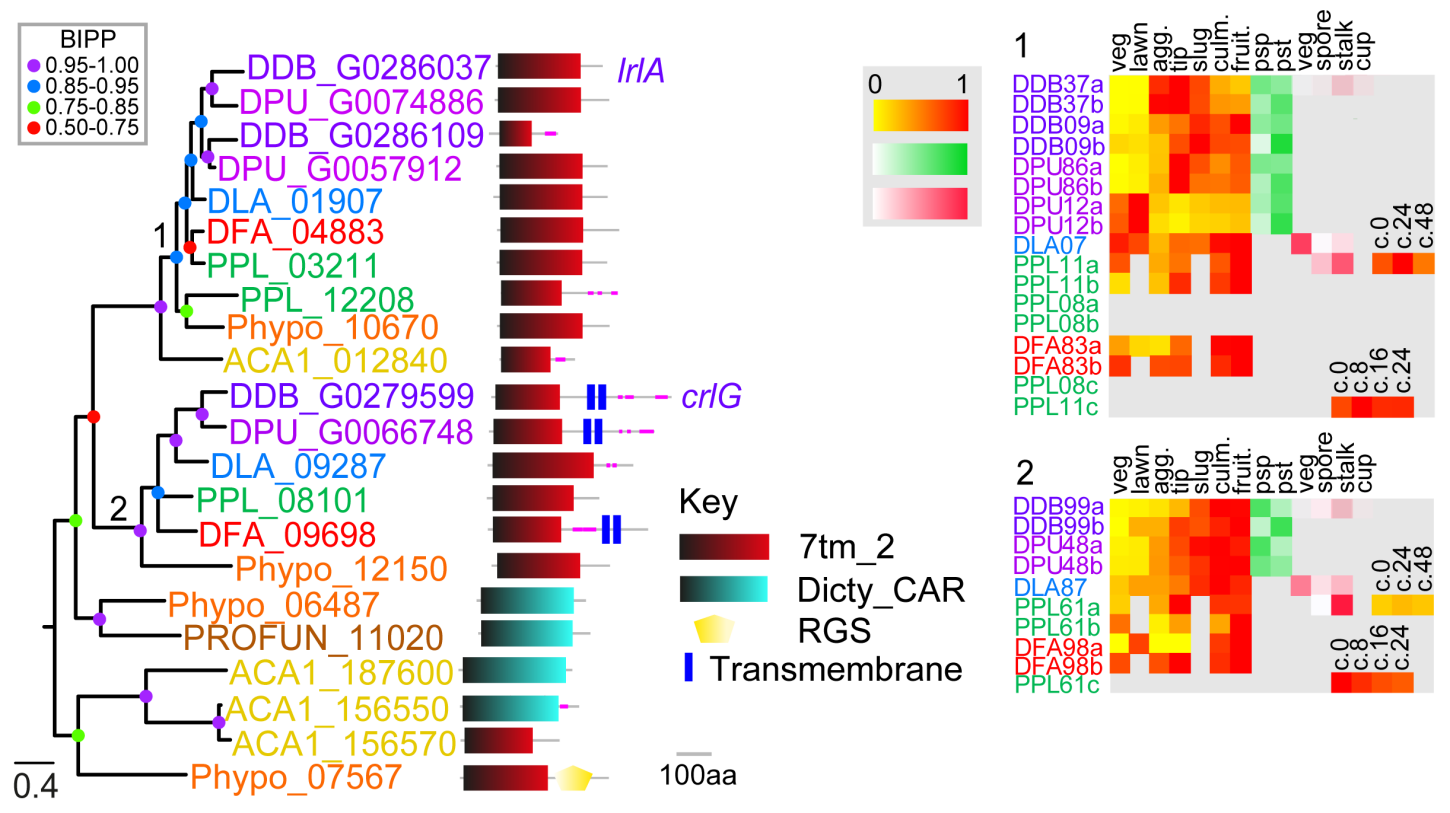
The family 2 secretin-like/Adhesion GPCRs. The family 2 GPCRs were identified by the presence of Interpro domain IPR000832 and by BLAST search and phylogenetic inference. A phylogeny was inferred from the final set of aligned proteins with MrBayes, which was annotated with the protein domain architectures and heatmaps of gene expression profiles as outlined in the legend to Figure 1. Note that clade 2 gene DDB_G0279599 was previously identified as a family 2 Car-like GPCR ( Prabhu & Eichinger, 2006), but we found it showed stronger phylogenetic affinity to family 2 GPCRs.

**Figure 3.**
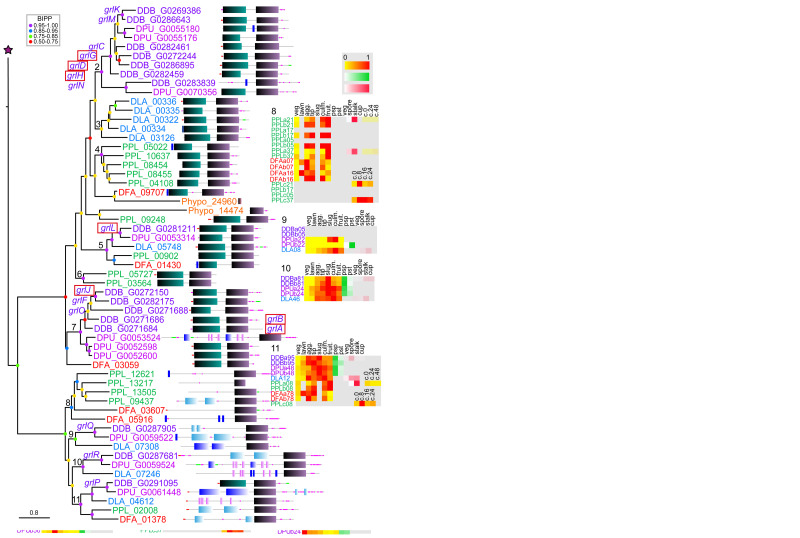
The family 3 metabotropic glutamate-like GPCRs. The family 3 GPCRs were identified by the presence of Interpro domain IPR017978 and by BLASTp search as outlined in the methods. A phylogeny was inferred from the final set of aligned proteins with MrBayes, which was annotated with the protein domain architectures and heatmaps of gene expression profiles as outlined in the legend to Figure 1. Apart from clade 1, GrlE, all other family 3 GPCRs form a single cluster of almost exclusively dictyostelid members. Other receptors of this family are only found in *Physarum polycephalum,* but not in other Amoebozoa.

**Figure 4.**
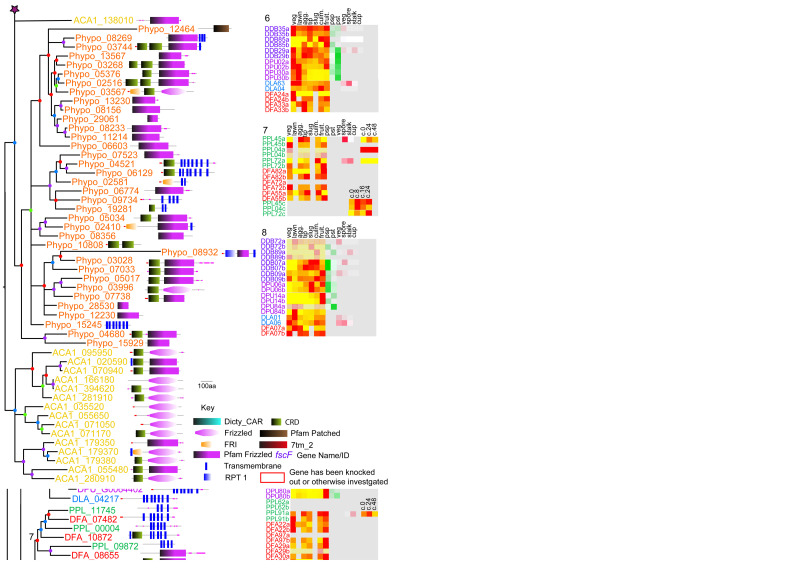
The family 5 frizzled-like GPCRs. The family 5 GPCRs were identified by the presence of Interpro domain IPR000539 and by BLASTp search as outlined in the methods. A phylogeny was inferred from the final set of aligned proteins with MrBayes, which was annotated with the protein domain architectures and heatmaps of gene expression profiles as outlined in the legend to Figure 1. The family 5 experienced independent amplifications in different lines of Amoebozoa and especially within group4 dictyostelids. The dictyostelid members all originate at the base of Dictyostelia and have no Amoebozoan orthologues.

**Figure 5.**
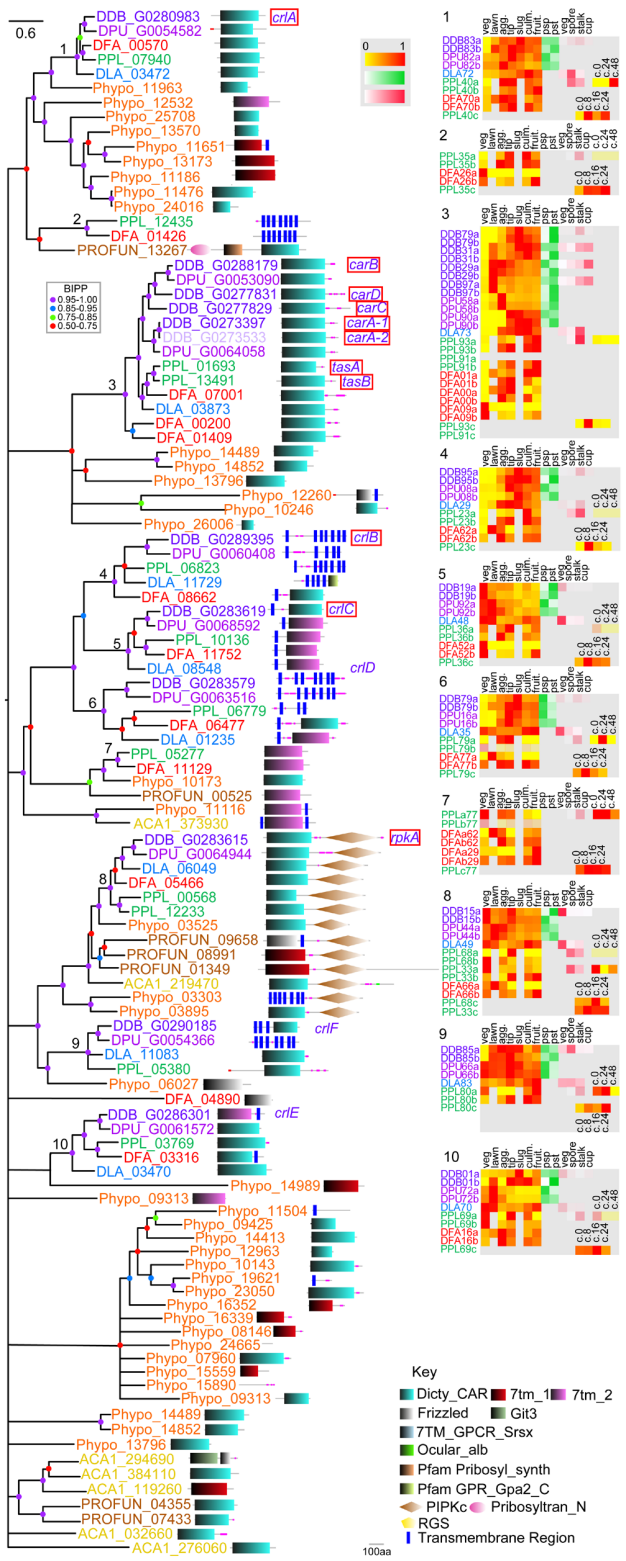
The family 6 cAR-like GPCRs. The family 6 GPCRs were identified by the presence of Interpro domain IPR000848 and by BLASTp search as outlined in the methods. A phylogeny was inferred from the final set of aligned proteins with MrBayes. Even after 3 million generations analysis did not converge and the deeper nodes of the tree are unresolved. Orthologous clades are, however, well defined. The tree was annotated with the protein domain architectures and heatmaps of gene expression profiles as outlined in the legend to Figure 1.

**Figure 6.**
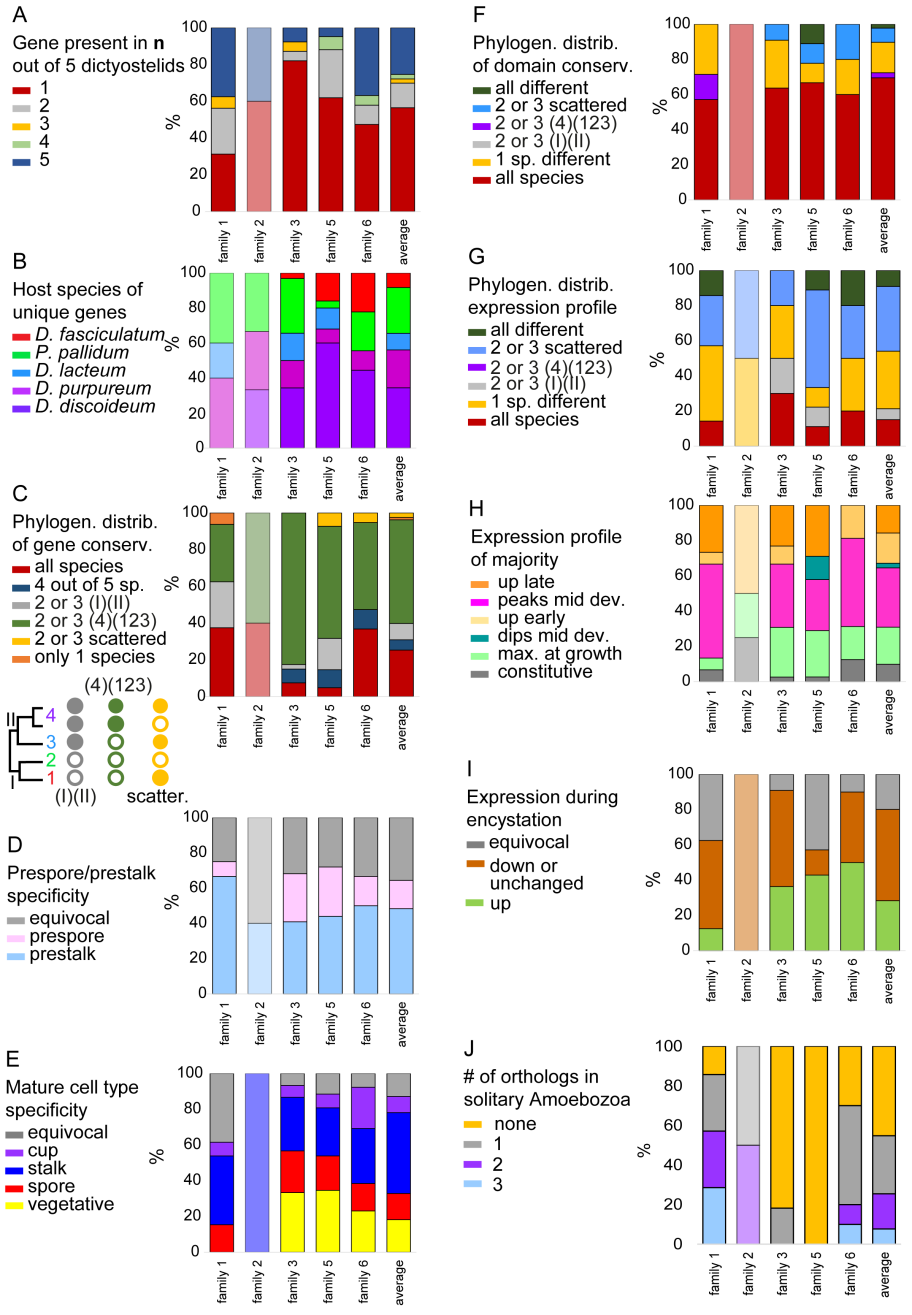
Summary of classed features across GPCR families. For each of the families we summarised and calculated the percentage of the different states of the following features: **A**. the total number of orthologs out of five species that were conserved for each gene. **B**. The host species of genes that were unique. **C**. The phylogenetic distribution of genes. **D**. The pre-cell (only for *D.dis* and *D.pur*) and **E**. mature cell type specificity (not for *D.fas*). The phylogenetic distribution of **F**. domain and **G**. expression profile conservation. **H**. The majority expression pattern during development. **I**. Expression tendency during encystation. (Data only available for *P.pal.*) **J**. Number of solitary Amoebozoan orthologues in clades. The name of each family is shown at the X-axis and the last bar represents the averages across all families. The figure is based on the data shown in Figure 1– Figure 5 that are compiled in supplemental file Compilation_GPCRs.xlsx and summarised in GPCR_traits.xlsx.

### Family 1: Rhodopsin-like GPCRs

19 subfamilies of rhodopsin-like GPCRs are recognized and grouped mainly by their type of ligands ( Joost & Methner, 2002). They are often involved in autocrine, endocrine and paracrine functions but a number of family 1 receptors are still orphan receptors with unknown ligand. Most rhodopsin-type GPCRs only contain a short *N*-terminus and bind their ligands inside the 7TM region. They are additionally membrane-anchored by palmitoylation of a conserved cysteine at the *C*-terminus of an additional eighth helix ( Hu *et al.*, 2017). No *C*-terminal palmitoylation motifs (apart from two low scoring ones in DFA_08662 and PHYPO_11504) were found in the Amoebozoan rhodopsin-like GPCRs using CSS-Palm palmitoylation site prediction ( Ren *et al.*, 2008). Rhodopsin-like receptors were not noted in an earlier study ( Prabhu & Eichinger, 2006), but six genes in *D.dis* were listed as “related to human transmembrane protein 145” and one as orphan receptor in a preceding comparative phylogenomic analysis ( Heidel *et al.*, 2011). Those six genes are forming clades 1 and 2 of the phylogenetic tree of family 1 in the present analysis. Nordström *et al.*, 2011 included atypical receptors and identified two GPR108-like and one IPR-like receptor in *D.dis* as separate branches. 

Phylogenetic inference subdivides the amoebozoan family 1 GPCRs into seven clades ( Figure 1). Apart from clade 5, which is missing group 4 orthologues, all clades show a complete set of dictyostelid orthologues from all five species. This high level of gene conservation differs from the other GPCR families. Exhaustive Blast searches failed to identify orthologues of clade 5 in *D.dis* or *D.pur* so, we must assume those genes were lost in the group 4 last common ancestor (LCA). All clades apart from clade 1 also contain at least one orthologue from either *A.cas*, *Pro.fun* or *Phy.pol.*, suggesting that these clades have deep ancestral roots in the solitary Amoebozoa, a notion that is also supported by the presence of a small clade of associated *Phy.pol* sequences at the root. Clade 1 shows extensive amplification of receptor genes in groups 2 and 4, with the group 4 duplicated genes present in both *D.dis* and *D.pur*, suggesting that the duplication event occurred in the LCA to group 4. Compared to the other GPCR families ( Figure 6D), the majority of dictyostelid family 1 GPCRs show a preference for expression in prestalk and stalk cells suggesting that these receptors might mediate effects of stalk-inducing signals. The receptors of clades 1 and 2 share their domain signature with animal rhodopsins whereas the other clades share domain similarity with the lung seven transmembrane receptors GPR107 and GPR108 (PF06814). GPR107 localises to the trans-Golgi network in human cells and is required for retrograde transport ( Tafesse *et al.*, 2014). GPR108 is also a trans-Golgi protein and required for Adeno-associated virus entry into the nucleus ( Dudek *et al.*, 2020). This subfamily also has homologues in fungi and plants and may have a conserved eukaryotic function in Golgi transport ( Edgar, 2007). We find typical bacterial rhodopsin-like domains only in the small outgroup of *Phy.pol* proteins. Unfortunately, no specific functions are known yet of rhodopsin family receptors in *Dictyostelium*.

### Family 2: Secretin-like/adhesion GPCRs

Family 2 GPCRs combines secretin-like and adhesion-type GPCRs ( Fredriksson *et al.*, 2003). The secretin-like GPCRs bind small peptides like secretin, calcitonin-gene related peptide, corticotropin-releasing factor, glucagon and glucagon-like peptides, growth hormone-releasing hormone, parathyroid hormone/parathyroid hormone-related peptides and vasoactive intestinal peptide which activate either the adenylyl cyclase pathway and/or the phosphatidyl-inositol-calcium pathway ( Lin *et al.*, 1991; Takei, 2016; Yang *et al.*, 2021). The secretin-like GPCRs usually contain a large extracellular *N*-terminal hormone-binding domain ( Yang *et al.*, 2021). Adhesion-type GPCRs mediate cell-cell and cell-extracellular matrix sensing and were identified as the ancestral type of this family ( Schiöth *et al.*, 2010). The adhesion-type GPCRs of animals, too, contain a large extracellular domain which is cleaved from the C-terminal domain by an interspersed GPCR auto-proteolysis inducing (GAIN) domain ( Vizurraga *et al.*, 2020). The GAIN domain encloses a tethered peptide-agonist stalk (also known as *Stachel* sequence) and dissociation of the *N*-terminal domain upon ligand binding leads to activation of the GPCR by back-folding of the liberated agonist-peptide ( Vizurraga *et al.*, 2020). A previous analysis revealed only a single *D.dis* secretin-like GPCR: Lathrophilin receptor-likeA (LrlA) ( Prabhu & Eichinger, 2006), which was also placed into the adhesion-type family by Schiöth ( Schiöth *et al.*, 2010). We found the Pfam GPCR family 2 domain signature (7tm_2) also in a clade comprising CrlG of *D.dis.* CrlG showed greater phylogenetic affinity to family 2 than to family 5 and was therefore included in this family here. Other dictyostelid GPCRs with the 7tm_2 domain showed a closer phylogenetic relationship to families 5 and 6 and were included there. No additional *N*-terminal domains or GAIN domains were detected in the family 2 GPCRs of Amoebozoa. BlastP searches with LrlA revealed the presence of such long N-terminal domains and the GAIN domain in Family 2 GPCRs of *Capsaspora owczarzaki*, a lineage within the opisthokonts closer to the origin of multicellular animals, but not in the latrophilin-like receptor A of *Hondaea fermentalgiana*, a member of the evolutionary distant stramenopiles ( Dellero *et al.*, 2018). This suggests that in the lineages leading to multicellular animals an ancestral simple eukaryotic receptor type was modified for new functions in cell-cell and extracellular matrix sensing by the addition of *N*-terminal and autoproteolysis domains. LrlA has undergone a gene duplication in group 4 dictyostelids, which for *D.dis* resulted in a truncated gene (DDB_G0286109) containing only four transmembrane domains ( Figure 2). We also detected simple adhesion-type receptors in the genomes of the three investigated solitary Amoebozoa. Nothing is known about the function of those receptors in *Dictyostelium* or other solitary Amoebozoa. Because of the small number of genes in this group, estimations of expression trends are difficult to interpret.

### Family 3: Metabotropic glutamate receptors/GABA receptors

The Family 3 of metabotropic glutamate and gamma-amino butyric acid (GABA _B_) receptors are only similar to other GPCRs on a structural level, but lack significant sequence similarity to them and probably represent an evolutionary separate group ( Fredriksson *et al.*, 2003). The family also contains Ca ^2+^-sensing (CaS), pheromone, taste and smell receptors ( Yang *et al.*, 2021). Ligand binding takes place in the *N*-terminus which forms a large extracellular domain. Family 3 GPCRs of vertebrates act as constitutive homo- or heterodimers ( Yang *et al.*, 2021). In accordance with previous studies ( Prabhu & Eichinger, 2006; Prabhu *et al.*, 2007b) we found 17 family 3 GPCRs in *D.dis* ( Figure 3). Many of the *D.dis* receptors resulted from gene amplifications specific to *D.dis* or group 4, while independent amplifications also occurred in groups 2 and 3 ( Figure 6A) or in groups 3 and 4 only ( *grlQ* and *grlR*). Only GrlE, GrlL and GrlP are fully conserved throughout the Dictyostelia with single orthologs per clade. The majority of family 3 GPCRs evolved exclusively in the dictyostelids with no homologues in the unicellular Amoebozoa ( Figure 3). Interestingly, GrlE has a number of related homologs in *Phy.pol* suggesting that this is the ancestral GABA receptor of the dictyostelids ( Figure 3). Similar to other family 3 GPCRs, most Amoebozoan Grl receptors exhibit a large *N*-terminal extracellular domain that shares similarity with either atrial natriuretic factor receptor (ANF; clade 1), basic membrane lipoprotein (BMP. clades 2-7) or has no recognised domains (clades 8-11). ANF is a secreted peptide involved in regulation of arterial blood and electrolyte balance in humans but also has functions in the central nervous system ( Stewart *et al.*, 1988). The phylogenetic tree of family 3 shows a significant dichotomy between GPCRs with and without the *N*-terminal ANF domain, with only GrlE belonging to the branch with ANF domains.

Biological roles for some *D.dis* Grls have been reported. GrlE mediates GABA activation of AcbA secretion from prespore cells ( Wu & Janetopoulos, 2013). AcbA is then processed by the TagA protease on prestalk cells to yield spore differentiation-factor 2 (SDF-2), which in turn induces maturation of prespore cells into spores ( Anjard & Loomis, 2006). This agrees with the observed cell-type specific expression of *grlE* in prespore and spore cells ( Figure 3). GrlG (Far2) and GrlL (Far1) both bind folate, but only GrlL was found to mediate folate regulated processes like chemotaxis ( Pan *et al.*, 2016). GrlH likely detects the chemorepellant chalone AprA, as *grlHˉ* cells phenocopy *aprAˉ* cells ( Tang *et al.*, 2018). GrlD is the putative receptor for extracellular polyphosphate, an inhibitor of cell proliferation ( Suess *et al.*, 2019). GrlJ is required for the correct timing of development and spore formation ( Prabhu *et al.*, 2007b), while *grlBˉ* cells exhibit delayed aggregation ( Wu & Janetopoulos, 2013). GrlA is required for proper late development and sporulation, probably mediating steroid-induced GABA release and subsequent SDF-2 production ( Anjard *et al.*, 2009; Prabhu *et al.*, 2007a). Overall, the observed expression patterns follow the reported place of action for the genes with known effects. It is remarkable that many of the Grls with established roles, except GrlE and GrlL (Far1), are the result of gene amplifications that only occurred in group 4, suggesting that these roles uniquely evolved in group 4 or its LCA. The GPCRs of family 3 show varied expression patterns but the highest level of conserved expression across taxon groups ( Figure 6G).

### Family 5: Frizzled/smoothened 7TMRs

Roles for frizzled/smoothened type receptors were first identified in animal development, where they are involved in tissue morphogenesis and patterning. Frizzled/smoothened receptors are defined by an *N*-terminal ligand binding, cysteine-rich domain (CRD) and a core seven-transmembrane domain which is terminated by the motif KTXXXW. They bind to extracellular ligands like Hedgehog and Wnts and mediate G-protein dependent and -independent signalling. The canonical Wnt/Frizzled signalling pathway acts via beta-catenin but frizzled receptors also function in the non-canonical planar cell polarity and Wnt/Ca ^2+^ pathways ( Huang & Klein, 2004). In Amoebozoa, frizzled-smoothened like (Fsl) receptors appear to have independently expanded from one or a few genes in *Phy.pol* and *A.cas*, but were not detected in *Pro.fun.* Dictyostelia also show extensive fsl gene amplification. Strikingly this particularly occurred in group 4, with most proteins found in *D.dis* ( Figure 4). Only a few dictyostelid frizzled-like GPCRs in clades 5, 7 and 8 contain the CRD domain indicating the loss would have occurred multiple times in receptor expansion in the dictyostelids, even though only clade 1 of nine *D.dis* proteins is referred to as the Frizzled/Smoothened-like Sans CRD (FscA-J). These proteins also have shorter *N*-termini ( Prabhu & Eichinger, 2006). Interestingly, Fscs only have homologues in *D.dis* and *D.pur* and most of the Fsc genes show developmental regulation. Of the remaining 14 *D.dis* Fsl receptors, several also lack a recognisable CRD domain even though longer *N*-terminal regions are present and only the *D.dis* Fsl receptors FslJ and FslK contain the conserved KTXXXW motif ( Prabhu & Eichinger, 2006). Most *A.cas* and *Phy.pol* receptors contain CRD domains, with the highest similarity scores to animal frizzled receptors found in *A.cas* ( Figure 4). Some dictyostelid Fsl GPCRs showed sequence similarity to the metazoan frizzled class receptors, but their SMART or PFAM frizzled domains were often not detected at E-values below the threshold of 0.001, indicating that they are considerably diverged.

Null mutants in *fscE*, *fslA*, *fslB* and *fslK* show defects in cytokinesis and responses to prestarvation signals, such as AprA ( Suess *et al.*, 2017; Tang *et al.*, 2018). In agreement with these early roles *fscE* and *fslB* are downregulated after growth, but *fslA* and *fslK* persist throughout development indicating additional roles there ( Figure 4). Like the family 1 rhodopsin-like receptors, the frizzled-like GPCRs (apart from clades 4 and 8) also show preferential expression in prestalk and stalk cells. Many family 5 GPCRs are also upregulated during encystation in *P.pal* and might perform signalling functions there.

### Family 6: Dictyostelid cAMP receptors (Dicty_CAR)

The signature cAMP receptor (cAR) of this family was first purified and identified from *D.dis* as the GPCR mediating chemotaxis to cAMP ( Klein *et al.*, 1987; Sun & Devreotes, 1991). Later, three lower affinity cAMP receptors and cAR-like receptors (Crl) were identified ( Louis *et al.*, 1994; Raisley *et al.*, 2004; Saxe *et al.*, 1991). The latter set did not bind cAMP and also had homologues outside Dictyostelia. The present analysis identifies a large number of Crl proteins in *Phy.pol* and somewhat fewer in *A.cas* and *Pro.fun*. ( Figure 5). The sequences that define the Dicty_CAR Pfam model overlap with those of other GPCR families (see *e.g.*, Figure 5, clades 7 and 9), but the overall sequence similarity of the proteins places them clearly within the Dicty_CAR family in phylogenetic analysis. While the deeper nodes of the phylogeny were not fully resolved in our analysis, we identified seven conserved clades that contain a full set of orthologs from dictyostelid species, with half of them also containing *Phy.pol* or *Pro.fun* orthologs or close homologs. Clade 1 contains a set of CrlA receptors including a *Phy.pol*. orthologue and a set of eight related *Phy.pol* proteins. CrlA was reported to detect the polyketide MPBD (4-methyl-5-pentylbenzene-1,3-diol), which stimulates both aggregation competence and release of the peptide SDF-1 (spore differentiation factor-1), which then promotes spore and stalk differentiation ( Anjard *et al.*, 2011). However, other workers found that CrlA does not mediate MPBD induction of aggregation competence and is not needed for normal spore differentiation in AX2 wild type cells ( Narita *et al.*, 2017). Clade 3 contains the four *D.dis* cAMP receptors (CarA, CarB, CarC and CarD). There are only two *D.pur* orthologues corresponding to CarA and CarB and separate duplication events have taken place in *P.pal* and *D.fas*. Cells in *D.dis* lacking the high affinity receptor CarA fail to aggregate and to express early aggregation genes ( Sun *et al.*, 1990). CarB and CarD have lower cAMP affinity and are expressed later in development, with CarB directing tip-oriented migration of prestalk cells ( Saxe *et al.*, 1991; Saxe *et al.*, 1993). The set of duplicated cARs in the group 2 species *P.pal* is required for post-aggregative morphogenesis and cAMP induction of prespore differentiation ( Kawabe *et al.*, 2009). Clade 3 also contains five *Phy.pol* GPCRs, which form an outgroup to the dictyostelid cAMP receptors. It is not known whether any of these bind cAMP. It would be intriguing to investigate whether at least one organism outside Dictyostelia uses cAMP as a secreted signal, or alternatively what the ancestral signal for the cARs might have been.

The cAMP receptor-like proteins CrlB, CrlC and CrlD are conserved throughout Dictyostelia and together form a well-supported group (clades 4–6). The *D.dis crlB* and *crlD* genes are expressed with a peak in mid-development, however their knockouts had no discernible phenotypes ( Raisley *et al.*, 2004). Whereas there were no solitary Amoebozoan orthologues of crlB-D, the larger grouping contains a clade (7) that consists of *P.pal* and *D.fas* (branch I), *Phy.pol* and *Pro.fun crl* genes. In addition, the grouping has a small basal clade of a *Phy.pol* and *A.cas crl* gene supporting an origin of the ancestral Crl of this clade in the Amoebozoa *.* It is worth noting that within this clade the domain sequences display a mixture of Dicty_CAR and 7TM_2 signatures, with the latter being more common for the secretin/adhesion GPCRs.

Clade 8 is made up of a full orthologous set of dictyostelid and amoebozoan Receptor Phosphatidylinositol Kinase A ( *rpkA*) genes, a *P.pal rpkA* duplicate and two additional *Phy.pol rpkA*-like genes ( Figure 5). The RpkA *s* consist of an *N*-terminal Dicty_CAR and a C-terminal phosphatidylinositol-4-phosphate 5-kinase (PIP5K) domain. The presence of clearly related homologues in all three solitary Amoebozoan species demonstrates that this domain arrangement was already present in the last common ancestor of all Amoebozoa. In *D.dis,* RpkA is involved in phagocytosis and in cell density sensing ( Bakthavatsalam *et al.*, 2006; Riyahi *et al.*, 2011). The related protein CrlF (clade 9) is present in most dictyostelids as well as *Phy.pol* but missing from *D.fas*. High BIPP support for a common origin of clades 8 and 9 points toward a split from an ancient receptor that happened before the root of the Amoebozoa. The pfam Frizzled/Smoothened domain was recognized at a lower E-value than the Dicty_CAR domain for clade 9, but phylogenetic inference showed strong support for its inclusion in the cAR-like family. *D.dis* CrlE (clade 10) is also well conserved throughout the Dictyostelia even though no homologues for CrlE could be found in other Amoebozoa and the position of the clade remains unresolved. There are additional large groupings of *Phy.pol, A.cas* and *Pro.fun* cAR-like receptors with no dictyostelid homologues and unclear phylogenetic affinities. Overall, the expression patterns of dictyostelid Dicty_CARs varied with a preference for being expressed in prestalk over prespore cells and in stalk cells ( Figure 6D, E).

## Conclusions

Compared to an earlier analysis ( Prabhu & Eichinger, 2006), some GPCRs have been assigned to different families and members of the Rhodopsin-like family have been identified for the first time in dictyostelids in this analysis. Receptor family diversification of original GPCRs is assumed to have taken place in the Amoebozoan ancestor ( Nordstrom *et al.*, 2011) and it is possible that those GPCRs have not acquired sufficient family distinctive signatures yet to be classed into the traditional GPCR scheme derived from animals using conventional similarity methods. Previous studies recognised six ( Heidel *et al.*, 2011) or three ( Nordstrom *et al.*, 2011) additional GPCRs in *D.dis*, that lie outside the classical six families.

The data from all five GPCR families has been quantified and summarised in Figure 6. We observe the highest degree of gene conservation in families 1 and 5 and the lowest in family 3, where gene amplification is seen across all groups ( Figure 6A). *D.dis* is overrepresented as host for unique genes indicating an overall higher degree of gene duplications in this species especially in Family 5 genes ( Figure 6B). In family 1, *D.pur* appears to be the host for unique genes, but this is due to small numbers of amplified genes. There is a general trend of gene amplification with group 4 species in all families ( Figure 6C).

The majority of GPCRs is more highly expressed in prestalk than prespore cells and this is particularly true for family 1 ( Figure 6D). Also, expression in stalk cells is higher than in spores for all families ( Figure 6E) The GPCRs individually show a range of different developmental expression profiles which is not very different between families. There is preference for peak expression in mid-development and only about ~20% of GPCRs are specifically expressed during growth. This seems to negate a notion that the many GPCRs of Dictyostelia may particularly be required for environmental sensing in the proliferative stage ( Figure 6E). 

Compared to the ~75% of GPCRs upregulated in multicellular development, less than ~30% are upregulated in encystation, with families 5 and 6 being most commonly upregulated. Combined, these observations suggest that the majority of *Dictyostelium* GPCRs contribute to the organisation of multicellular development.

GPCR families 1, 2 and 6 already diverged early in the Amoebozoa demonstrated by a larger number of orthologues in two or three of the solitary species, whereas families 3 and 5 mostly diverged within the dictyostelids. Most of the functionally analysed *D.dis* members of the Frizzled/Smoothened-like receptors have roles during growth. Our analysis identified homologues of this family which are developmentally upregulated and are worthy of further study. Likewise, the functions of the newly identified family 1 GPCRs are as yet unexplored. It could well be possible that this family harbours the yet unidentified dictyostelid photoreceptor.

## Data Availability

All data files have been deposited within an OSF registry entitled “G protein coupled receptors of dictyostelids” under DOI 10.17605/OSF.IO/AP4NJ under license CCO 1.0 Universal. The Excel spreadsheet Social_Amoebas_Expression.xlsx is mostly identical to Additional file 2: Table S1 in ( Forbes *et al.*, 2019) with addition of the *D.pur* genomic locus tags. This project contains the following underlying data: Social_Amoebas_Expression.xlsx (combined RNAseq expression data of social amoebas from several sources) GPCRs_expression.xlsx (Raw and normalised expression data for GPCRs from social amoebas) Compilation_GPCRs.xlsx (Family by family enumeration of traits by clades) GPCRs_traits.xlsx (summarised trait data for all GPCR families from Compilation_GPCRs.xlsx used for generation of summary Figure 6) GPCRfam1.fas (Fasta file of all GPCR family 1 protein sequences) GPCRfam1.nex (edited alignment file for all GPCR family 1 sequences in nexus format) GPCRfam2.txt (Fasta file of all GPCR family 2 protein sequences) GPCRfam2.nex (edited alignment file for all GPCR family 2 sequences in nexus format) GPCRfam3.fas (Fasta file of all GPCR family 3 protein sequences) GPCRfam3.nex (edited alignment file for all GPCR family 3 sequences in nexus format) GPCRfam5.txt (Fasta file of all GPCR family 5 protein sequences) GPCRfam5.nex (edited alignment file for all family 5 sequences in nexus format) GPCRfam6.txt (Fasta file of all GPCR family 6 protein sequences) GPCRfam6.nex (edited alignment file for all GPCR family 3 sequences in nexus format) Data are available under the terms of the Creative Commons Zero “No rights reserved” data waiver (CC0 1.0 Public domain dedication). The raw RNA-Seq data of Dlac cell types and Ppal encystation time series are available from Arrayexpress https://www.ebi.ac.uk/biostudies/arrayexpress/studies/E-MTAB-7824?query=Dictyostelium%20lacteum under accession number E-MTAB-7824. The other RNAseq expression data were obtained from published sources as indicated.
