# Supplementary material for: Phylogeny-wide analysis of G-protein coupled receptors in social amoebas and implications for the evolution of multicellularity
Source: Open Res Eur. 2023 Feb 15;2:134. Originally published 2022 Dec 6. [Version 2] doi: 10.12688/openreseurope.15250.2 (PMC10445921; doi:10.12688/openreseurope.15250.2)
Supplement: Peer review history

# Peer review: Reviewer response for version 2

**DOI:** 10.21956/openreseurope.16865.r30850

**Published:** 2023-02-16

**Authors:** Jeffrey A. Hadwiger

**Affiliations:** 1 Department of Microbiology and Molecular Genetics, Oklahoma State University, Stillwater, OK, USA

The authors have addressed all of my comments.

Is the study design appropriate and does the work have academic merit?

Yes

Is the work clearly and accurately presented and does it cite the current literature?

Yes

If applicable, is the statistical analysis and its interpretation appropriate?

Yes

Are all the source data underlying the results available to ensure full reproducibility?

Yes

Are the conclusions drawn adequately supported by the results?

Yes

Are sufficient details of methods and analysis provided to allow replication by others?

Yes

Reviewer Expertise:

Signal transduction, chemotaxis, development, amoeba, G proteins, receptors, MAP kinases, and phosphodiesterases

I confirm that I have read this submission and believe that I have an appropriate level of expertise to confirm that it is of an acceptable scientific standard.

# Peer review: Reviewer response for version 2

**DOI:** 10.21956/openreseurope.16865.r30851

**Published:** 2023-02-15

**Authors:** Xuehua Xu

**Affiliations:** 1 Chemotaxis Signaling Section, Laboratory of Immunogenetics, National Institute of Allergy and Infectious Diseases, National Institutes of Health (NIAID/NIH), Rockville, MD, USA

The manuscript is suitable for indexing.

Is the study design appropriate and does the work have academic merit?

Yes

Is the work clearly and accurately presented and does it cite the current literature?

Yes

If applicable, is the statistical analysis and its interpretation appropriate?

Yes

Are all the source data underlying the results available to ensure full reproducibility?

Yes

Are the conclusions drawn adequately supported by the results?

Yes

Are sufficient details of methods and analysis provided to allow replication by others?

Yes

Reviewer Expertise:

GPCR functions in diverse processes

I confirm that I have read this submission and believe that I have an appropriate level of expertise to confirm that it is of an acceptable scientific standard.

# Peer review: Reviewer response for version 1

**DOI:** 10.21956/openreseurope.16492.r30522

**Published:** 2023-02-03

**Authors:** Xuehua Xu

**Affiliations:** 1 Chemotaxis Signaling Section, Laboratory of Immunogenetics, National Institute of Allergy and Infectious Diseases, National Institutes of Health (NIAID/NIH), Rockville, MD, USA

Authors analyzed phylogenetic distribution and domain composition, and their temporal/spatial expression of GPCRs in five species of dictyostelids. The work is informative and will provide essential information for future study.

 Minor points:

1. It would be helpful for readers to briefly mention the known ligands and/or functions of GPCRs, such as GPR108 or IPR-like receptor when they were first mentioned the manuscript.
2. It would be very helpful to discuss a little more on the similarity and differences of the rhodopsin-like receptors between dictyostelids and their bacterial or animal rhodopsins.
3. Labels for figures are generally too small to read, especially those in the heat map of expression. Figure 2 is good.

Is the study design appropriate and does the work have academic merit?

Yes

Is the work clearly and accurately presented and does it cite the current literature?

Yes

If applicable, is the statistical analysis and its interpretation appropriate?

Yes

Are all the source data underlying the results available to ensure full reproducibility?

Yes

Are the conclusions drawn adequately supported by the results?

Yes

Are sufficient details of methods and analysis provided to allow replication by others?

Yes

Reviewer Expertise:

GPCR functions in diverse processes

I confirm that I have read this submission and believe that I have an appropriate level of expertise to confirm that it is of an acceptable scientific standard, however I have significant reservations, as outlined above.

# Response

**Published:** 2023-03-08

**Authors:** Christina Schilde

**Affiliations:** University of Dundee, UK

Dear Reviewer 2, thank you for taking the time to review our article and for pointing out some omissions and errors. Your critical reading helped to improve the article. Minor points:

1. **"It would be helpful for readers to briefly mention the known ligands and/or functions of GPCRs, such as GPR108 or IPR-like receptor when they were first mentioned the manuscript."**
  We have added information about the function of GPR108 and IPR.
2. **"It would be very helpful to discuss a little more on the similarity and differences of the rhodopsin-like receptors between dictyostelids and their bacterial or animal rhodopsins."**
  We have added a brief discussion of the similarity to animal and bacterial rhodopsins. We have also inserted an additional section into the conclusions.
3. **"Labels for figures are generally too small to read, especially those in the heat map of expression. Figure 2 is good."**
  We appreciate the labels are too small and have tried to replace them by letter labels where only every other character is shown in order to improve readability because this was also an issue the other reviewer had raised. We hope that this has improved the figures.

# Peer review: Reviewer response for version 1

**DOI:** 10.21956/openreseurope.16492.r30523

**Published:** 2023-01-13

**Authors:** Jeffrey A. Hadwiger

**Affiliations:** 1 Department of Microbiology and Molecular Genetics, Oklahoma State University, Stillwater, OK, USA

The authors have conducted an extensive comparison of GPCRs in amoeba and produced family specific phylogenetic trees for each of the major GPCR families. The designation of new GPCRs and phylogenetic relationship of GPCRs should prove insightful for those studying GPCRs and amoeba. The analysis is based on existing genomic and expression data from several amoeba species including developmental and cell-type specific expression. Overall this report is well written and easy to understand but there are some clarifications that could be useful to the readers (see comments below).

- Several abbreviations are not defined.

For example GPR and IPR  (InterPro terminology?).

 A supplementary table with the full names of the abbreviated receptors and other designations. in the figure keys would help with understanding what is being referenced.

 It is unclear what the c.0-c.24 refers to – counts? More explanation needed.

- The heat map labels on the X-axis are difficult to read – not sure of a remedy other than zooming in.
- The expression data in general appears to be weak and inconsistent in some cases between duplicate experiments. These inconsistencies are due to the original data source but the authors might caution the readers about the validity of this information.
- In the first paragraph describing family 6, the sentence “The latter set [referring to Crl receptors] did not bind cAMP and . . . “ The ability to bind cAMP was never tested for these receptors based on the recollection of this reviewer.
- The BIPP color coding key does not include yellow junctions. The significance of the yellow junctions should be noted.
- Fig. 4 appears to be cut off on the bottom

Is the study design appropriate and does the work have academic merit?

Yes

Is the work clearly and accurately presented and does it cite the current literature?

Yes

If applicable, is the statistical analysis and its interpretation appropriate?

Yes

Are all the source data underlying the results available to ensure full reproducibility?

Yes

Are the conclusions drawn adequately supported by the results?

Yes

Are sufficient details of methods and analysis provided to allow replication by others?

Yes

Reviewer Expertise:

Signal transduction, chemotaxis, development, amoeba, G proteins, receptors, MAP kinases, and phosphodiesterases

I confirm that I have read this submission and believe that I have an appropriate level of expertise to confirm that it is of an acceptable scientific standard, however I have significant reservations, as outlined above.

# Response

**Published:** 2023-02-06

**Authors:** Christina Schilde

**Affiliations:** University of Dundee, UK

Dear reviewer 1, Thank you for taking the time to review our article and for pointing out some omissions and errors. Your critical reading helped to improve the article.

- ***"Several abbreviations are not defined."***

Thank you for the suggestion. We have inserted a table of abbreviations for all domains and receptors at the end. The names of the human genes GPR108 (G-protein-coupled receptor 108) and ITR-like (Intimal-hyperplasia-related gene-like) are derived from the HUGO (human gene nomenclature committee) terminology. IPR numbers are indeed Interpro domain identifiers. The c.0-c.48 are reads from encystation time courses of PPL and were explained in the figure legend to Figure 1. We have replaced those labels with small letters and have explained them in the figure legend to Figure 1.

- ***"The heat map labels on the X-axis are difficult to read – not sure of a remedy other than zooming in."***

We appreciate the labels are too small and have tried to replace them by letter labels where only every other character is shown in order to improve readability because this was also an issue the other reviewer had raised. We hope that this has improved the figures.

- ***"The expression data in general appears to be weak and inconsistent in some cases between duplicate experiments. These inconsistencies are due to the original data source but the authors might caution the readers about the validity of this information."***

Expression data with extremely weak read counts (<10) have already been indicated shaded in 30% opacity. Of course, we could raise the threshold, but we also sometimes observe problems between the duplicate datasets even with higher read counts. We have inserted a sentence at the end of the methods section cautioning the readers about incongruent duplicate datasets.

- ***"In the first paragraph describing family 6, the sentence “The latter set [referring to Crl receptors] did not bind cAMP and . . . “ The ability to bind cAMP was never tested for these receptors based on the recollection of this reviewer."***

This was an overinterpretation of the fact that all Crl strains aggregate normally. We have reworded the sentence.

- ***"The BIPP color coding key does not include yellow junctions. The significance of the yellow junctions should be noted."***

Thanks for spotting this oversight. Yellow junctions only appear in Figure 3 as this was the only tree that could not be resolved with Bayesian phylogeny and hence was produced with RAxML. We have replaced the inset legend with a corrected legend and also corrected the figure legend.

- ***"Fig. 4 appears to be cut off on the bottom"***

Again, this was an oversight and you thankfully spotted this. A small number of clades was indeed cut off at the bottom of Figure 4 in the production of the PDF file. A complete Figure 4 is now attached.
